# A novel sensor-based digital instrument for assessment of quality of fibre extracted from banana pseudostem

**DOI:** 10.1016/j.heliyon.2024.e37155

**Published:** 2024-08-30

**Authors:** Deb Prasad Ray, Prateek Shrivastava, Rakesh Kumar Ghosh, Manik Bhowmick, D.B. Shakyawar, Ipsita Das, Gunasindhu Sardar, Jayanta Mondal, S.C. Saha, Gautam Roy

**Affiliations:** a ICAR-National Institute of Natural FIbre Engineering and Technology, Kolkata, 700040, India; b College of Agriculture, Auburn University, AL, AL, 36849, USA

**Keywords:** Banana pseudostem, Fibre, Sensors, Microcontroller, And HMI

## Abstract

Banana (*Musa paradisiaca*) farming generates huge quantities of biomass, all of which goes to waste due to the non-availability of suitable technology for its commercial application. The potential solution to this issue could be the conversion of pseudo-stems into valuable assets by converting them into fibres for various textile and non-textile applications. The specific characteristics of banana pseudo-stem fibre i.e. high absorptivity, breathability and biodegradability made it sustainable as well as suitable for the development of diversified products and blending with other natural fibres. However, non-uniformity in availability, obscurity of its intended uses and lack of knowledge for assessment of fibre quality posed a biggest hurdle to reach the fibre into the textile markets. Hence, a novel sensor-based digital instrument for assessing the quality parameters i.e. bundle strength and fineness along with overall grade of banana pseudo-stem fibre is presented in this research article. The developed instrument mainly consists of a fibre bundle strength measurement unit, fineness measuring unit and visual interface cum data acquisition unit. Test results indicated that bundle strength and fineness measured by developed instrument varied from 20.92 g/tex to 28.31 g/tex and 5.63 tex to 6.41 tex respectively. Furthermore, a good correlation between the measured and actual outputs of bundle strength (*One-Way ANOVA, F*_*28,2*_ = *3.914, P* = *0.224*), fineness (*One-Way ANOVA, F*_*51,2*_ = *4.730, P* = *0.190*) and overall quality of fibre (*Independent sample T-Test, F*_*34,1*_ = *0.95, P* = *0.190*). Was observed at 5 % level of significance. The present study also introduced a grading system for quality assessment of banana fibre based on the well-established and well-recognized grading system of jute fibre developed by Indian Standard (IS: 271 2020). The developed instrument is easy to build as well as easy to use and have an approximate cost of $1800.00. The combination of developed instrument and grading system is an accurate, feasible and time-ordered technique for the assessment of the overall quality of the banana fibre and well suited for the actual conditions.

## Abbreviations

$: US dollar
ADC: Analog-to-digital convertor
CAD: Computer Aided Design
db: Dry Basis
DC: Direct Current
g: Gram
GUI: Graphical User Interface
H: Height
HMI: Human Machine Interface
IDE: Integrated Development Environment
ICAR: Indian Council of Agricultural Research
IC: Integrated Circuit
IS: Indian Standard
kg: Kilogram
kPa: Kilo Pascal
L: Length
mg: Mili gram
NINFET: National Institute of Natural Fibre Engineering and Technology
PCB: Printed Circuit Board
RH: Relative Humidity
SD: Secure Digital
V: Volt
W: Width

## 1 Introduction

The scientific community's recent emphasis has shifted towards environmental preservation and sustainable industrial practices in response to escalating global environmental concerns. This shift entails strategically integrating naturally occurring and renewable materials into green manufacturing processes [[1], [2], [3], [4]]. The growing need for sustainable materials in various industries has led to a heightened demand for alternatives to forest-based raw materials and synthetic polymers. One particular category of sustainable materials is agro-waste residues, which are being investigated by researchers for their potential industrial applications [4,5]. One valuable application of agricultural waste lies in the textile industry [6], as it serves as a rich source of natural fibres. These fibres represent environment-friendly alternatives to synthetic fibres [5]. The superiority of natural fibres compared to synthetic fibres can be attributed to their exceptional comfort, favorable mechanical characteristics, natural abundance, cost-effectiveness, acoustic attributes, and biodegradability [[10], [11], [12], [13], [7], [8], [9]]. Therefore, in recent years, there has been an increasing adoption of renewable and environment-friendly natural fibres has been advocated by the textile industries as a response to the diminishing reserves of fossil fuels and ongoing environmental degradation [14]. Among various natural fibres, cotton and silk have already gained significant attention in textile industries. However, the conventional cultivation practice of cotton has raised concerns about its sustainability due to the extensive use of fertilizers as well as pesticides and high water consumption, which adversely affects the soil quality and the surrounding ecosystem. Similarly, the economic viability of using silk, an animal-derived fibre, is not always favorable. In response, agro-residues-based fibre such as bananas have emerged as promising alternatives due to their abundant availability and potential to address these concerns [15]. The fibres of the banana can be extracted from the pseudo-stem (trunk) or the bunch (peduncle). These agro-waste materials are widely accessible in most regions of the world [16,17].

India is the largest producer of banana across the globe having a total cultivation area of around 0.96 million ha with a production of around 36.6 million tons [18]. The geographical condition of India is congenial for cultivation of banana but it is predominantly grown in Andhra Pradesh, Karnataka, Maharashtra, Kerala, Tamil Nadu and West Bengal states of the country. Banana farming generates huge quantities of biomass all of which goes to waste due to non-availability of suitable technology for its commercial utilization [19]. A single hectare of banana plantation yields approximately 220 metric tons of waste [20]. Worldwide approximately 1.2 billion tons of waste is generated every year due to banana pseudo-stems [4] whereas India alone produces about 190 million tons of biomass waste [5]. The disposal of banana waste, specifically the pseudo-stem, contributes significantly to the release of substantial amounts of methane gas and carbon dioxide into the atmosphere during decomposition. Additionally, the burning of banana pseudo-stem is also environmentally detrimental, with reports indicating that the burning of one ton of pseudo-stem waste produces half a ton of carbon dioxide [21]. Hence, efficient management of such quantity of banana pseudo-stems in both sustainable and lucrative manner is one of the biggest challenges in the Indian agriculture system [22,23]. The potential solution to this issue could be the conversion of pseudo-stems into valuable assets [24] by converting them into fibres in bulk quantities through the extraction process [[25], [26], [27]]. The application of banana pseudo-stems waste-based fibres in textile sectors [6,28,29] can also help in reducing the pile-up of unmanaged agro-waste with minimum to no risk of environmental pollution [30]. The reclamation of this deposed banana waste also has the potential to promote a circular economy [31], to strengthen the economies of the banana fruit-cultivating nation [29,32] like India. It was reported that approximately 400 kg fibres per hectare can be extracted from the banana pseudo-stem waste and can help to create USD 3.5 to 4.8 billion of the market in the Indian textile sector [22,27]. Farmers stand to gain financially by selling their banana plant wastes, while textile manufacturers will profit from the abundance of inexpensive and enticing raw materials, resulting in increased production volumes and higher profits. Furthermore, this scenario will lead to additional benefits such as job creation and heightened exports attributable to reduced production costs [4,33].

Fibre obtained from Banana pseudo-stem has immense potential for its diversified applications in textile and non-textiles sector [5,[34], [35], [36], [37], [38], [39], [40], [41], [42], [43]]. The specific characteristics of banana pseudo-stem fibre i.e. high absorptivity, breathability and biodegradability made it sustainable as well as suitable for the development of diversified products and blending with other natural fibres [[44], [45], [46], [47], [48], [49]]. Due to its excellent characteristics, banana fibre has emerged as an alternative to cotton fibre for apparel production in recent years [50]. In addition to this, increasing cost of synthetic fibres like glass, carbon, plastics, asbestos, etc., in the international market and the health risks caused during their manufacturing and processing [51] opened a flattering and appealing option for banana fibre because of its renewability, recyclability, economic effectiveness, and environmental friendliness [11,27,52].

Despite immense potential, banana fibre is still not very popular in the Indian textile market, due to the obscurity of its intended uses. Research related to the selection of fibre in textile production is mostly confined to the laboratory and stakeholders still have not benefited from that. Furthermore, banana fibre is also heterogeneous in quality and varies from place to place according to the agroclimatic conditions and extraction processes adopted by the farmers [53]. Therefore, technologies for identification as well as segregation of fibre in terms of quality is a need of the hour for researchers, academia and stakeholders.

Recent developments in computer and electronics technology have made measurements of various quality parameters of natural fibres easier. Hence, the development of sensors and transducers-based instruments for such purposes has increased the attention of researchers. As of now, no substantial research pertaining to the assessment of quality of banana fibre has been evidenced around the globe. But a few pieces of literature are sporadically available for other natural fibres. For measurement of individual properties of natural fibre i.e. bundle strength [54]and fineness [[55], [56], [57]] efforts were taken by the researchers. Roy et al. (2017) [58] and Ghosh and Das (2013) [59] also worked on the measurement of multiple quality parameters and the overall grade of jute fibre simultaneously but this cannot be applicable to banana fibre.

This necessitates the development of an advanced sensor-based instrument that can be used for the assessment of quality of banana fibre. Therefore, the present study was aimed at the objective of development of sensors, microcontrollers and HMI-based banana fibre quality assessment instrument. The instrument was also validated in actual conditions to analyze the quality of fibre of different cultivars grown in different agroclimatic zones in India.

## 2 Theoretical consideration

### 2.1 Fibre bundle strength

The strength is generally defined as the ability of the fibre to resist the maximum tension to the limit of rupture. The strength of the fibre bundle is expressed in tenacity and can be represented mathematically using equation (1).

(1) $$\tau_{f}=\frac{F\times l}{m}$$

Where;

$\tau_{f}$ = tensile strength/tenacity of the fibre, g/tex; F = force required to break the bundle of fibres, kg; l = length of the fibre sample, mm and m = mass of the fibre sample, mg

### 2.2 Fibre fineness

Fineness is defined as the average diameter of the equivalent circle made by the single filament of the fibre and is mathematically represented by using equation (2). More recently the tex system has been adopted for the measurement of the fineness of fibres and yarns. The tex is the grams per kilometre or milligrams per meter of the fibre and can be mathematically represented by equation (3).

(2) $$D_{f}=\sqrt{\frac{4m}{\pi \;\rho \;l}}$$

(3) $$f_{tex}=\frac{M}{L}$$

Where;

$D_{f}$ = average diameter of the single fibre filament, mm; m = mass of the fibre sample, mg; ρ = density of the fibre sample, mg/mm^3^; l = length of the fibre sample, mm; $f_{tex}$ = Fibre fineness, tex; M = mass of the single fibre filament, g and L = total length of single fibre filament, km

### 2.3 Defects

The major defects in banana fibre are the content of good amount of adhered short fibres, or some number of gummy substances which do not contribute either for strength, nor in the fineness of the fibre. Defect of the banana fibre is expressed in terms of per cent by weight of the samples mass.

### 2.4 Colour

The property of the fibre which distinguishes its appearance as redness, yellowness, greyness etc [60].

### 2.5 Debris content

These are dust and mud, moss and sticks which are mostly lost during processing and are thus subject to claims [61].

## 3 Materials and methods

As banana fibre is a kind of lignocellulosic fibre having similar mechanical as well as physical properties to jute fibre [[62], [63], [64], [65], [66]], therefore, the methodology and prerequisites as reported by Roy et al., 2009 [54], Roy et al., 2017 [58], Saha and Bhaduri 2008 [67]; and Saha et al., 2022 [68] employed for the development of system for measurement of banana fibre bundle strength whereas concepts reported by Roy et al., 2013 [56], Roy et al., 2017 [58] and Sinha and Bandyopadhyay 1968 [69]; was employed for the development of system for measurement of fineness of fibre. The comprehensive description of the developed instrument is enumerated in the subsequent sections.

### 3.1 Architecture of the instrument

**Fig. 1:**
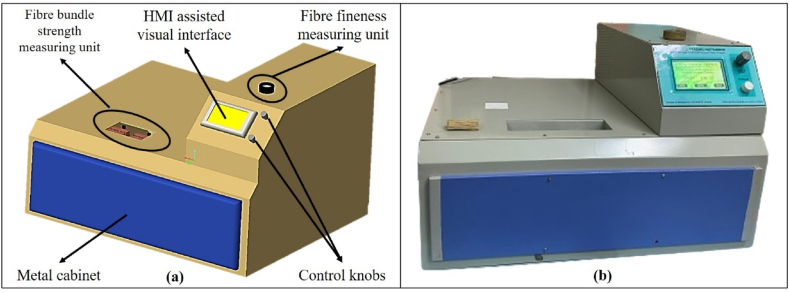
(a) CAD view and (b) developed view of banana fibre quality assessing instrument.

For assessing the quality of banana fibre, a microcontroller as well as sensor-based digital instrument was designed and developed. The CAD view and the developed view of the instrument are shown in Fig. 1 (a) and (b) respectively. The developed instrument mainly consists of a fibre bundle strength measurement unit, fineness measuring unit and visual interface cum data acquisition unit. Entire unit was housed in a specially developed metal cabinet for safe and proper operation of the instrument. The instrument has overall dimensions of L × W × H = 510 mm × 610 mm × 370 mm.

### 3.2 Fibre bundle strength measurement unit

**Fig. 2:**
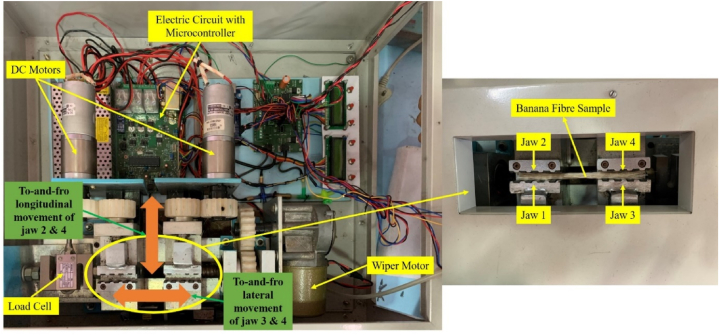
Hardware of the developed fibre bundle strength measurement system.

For measuring the breaking strength of fibre, an embedded unit as shown in Fig. 2 was developed. The developed unit was composed of three sub-units i.e. banana fibre holding sub-unit, breaking strength measuring sub-unit and data processing sub-unit. Banana fibre holding sub-unit consists of two sets of jaw vice for griping or holding fibre sample, two 12 V 300 rpm DC geared motor (RKI 1188) for longitudinal to-and-fro movement of jaw 2 and 4, one 12 V DC operated wiper motor (PGW-8007) for lateral to-and-fro movement of jaw 3 and 4 combinedly, three 2-Channel 5 V relay modules, three current sensors (ACS712-20A) and ramp generator circuit. Breaking strength measuring sub-unit includes an S-type load cell (Manufacturer: EPOCH Instruments & Controls Pvt Ltd, Model: LZYB, Capacity: 200 kg) and 24-Bit Analog-to-Digital Converter (HX711), whereas data processing sub-unit includes an ATmega 32 microcontroller.

For placing the fibre sample in banana fibre holding sub-unit for bundle strength measurement as illustrated in Fig. 2, jaws 2 and 4 were moved individually to-and-fro in a longitudinal direction with the help of two DC motors (one motor for movement of one jaw). For to-and-fro movement of the jaws in a longitudinal direction, one 2-Channel 5 V relay module, and one current sensor (ACS712-20A) were integrated with both DC motors. Once the fibre was gripped properly, jaws 3 and 4 were moved simultaneously to-and-fro in a lateral direction through nylon-made gear mechanism for tearing off the fibre sample. For this lateral movement of the jaws, an electronic ramp generator circuit was developed by using the 2-Channel 5 V relay module, current sensor (ACS712-20A), capacitor, bipolar transistor and junction field effect transistor (JFET). This ramp generator was connected in series with the wiper motor, to produce the linearly increasing current with respect to the time and having a constant slope. The mathematical relation between the current and torque generated by the DC motor is expressed by equation (4).

(4) $$T=f\left(\emptyset .I_{A}\right)$$

where, ∅ = flux per pole, weber; and $I_{A}$ = armature current, A

As a permanent magnet type DC motor with ramp generator was used in the present work, therefore, flux (∅) remains constant and can be expressed mathematically by equation (5).

(5) $$T\propto I_{A}$$

According to the mathematical relation, torque induced in the motor was proportional to the current utilized by the motor. Therefore, the ramp generator induced a linearly increasing torque to the motor which was fed to the arm. This applied torque was multiplied by the ratio of length from the pivot to the motor end with the length from the pivot to the fibre holding end. Hence, induced force on the fibre bundle was increased at a constant rate. A limit switch was also provided in connection with the ramp generator circuit to limit the movement of wiper motor. Once the fibre sample was teared off, limit switch turned off the ramp generator circuit and cut off the current supply of motor to stop the lateral movement of the motor. At this instant, the value of the current was latched and the latch circuit stored this value. This stored value of the current taken by the motor was converted into the force induced in the motor by using equations (4), (5)).

Once the fibre sample was torn off, the S-type load cell used in the breaking strength measuring sub-unit sensed the breaking load of the fibre in terms of analog values. To convert the analog values into digital values a 24-Bit Analog-to-Digital Converter (HX711) was connected in series with the load cell.

**Fig. 3:**
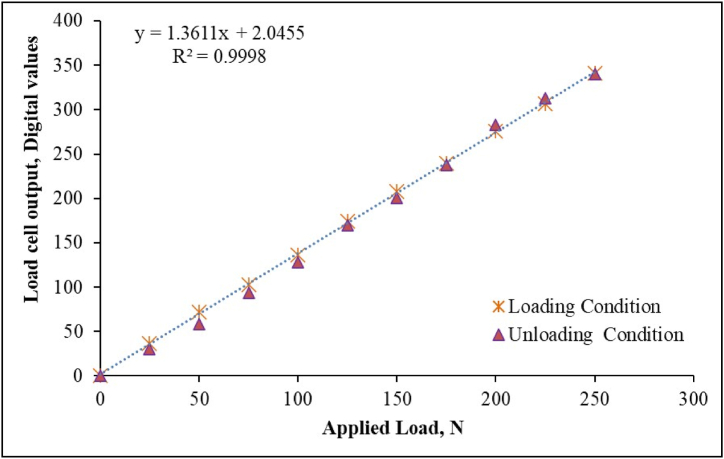
Calibration curve of load cell.

Before installing the load cell into developed unit, it was calibrated in the laboratory, the calibration curve as illustrated in Fig. 3 represents a very good colinear relationship (R^2^ = 0.99) between the applied load and load cell output.

After that, digitally processed data of the load cell was transmitted to the ATmega 32 microcontroller of the data processing sub-unit. The microcontroller further processed the data and converted it into the desired form of bundle strength in terms of tenacity by using the mathematical relation as given in equation (6). To control the lateral and longitudinal operation of all three motors along with the conversion of load cell output into fibre bundle strength in terms of tenacity (equation (7)), an algorithm was developed in C^++^ language by using the Arduino IDE and uploaded on the microcontroller.

(6) $$\tau_{f}=125\times \left(\frac{F_{f}}{W_{f}}\right)$$

(7) $$\tau_{f}=125\times \left(\frac{F_{f}}{\left\{\left(1.3611\times x\right)+2.0455\right\}/9.81}\right)$$

Where: $\tau_{f}$ = Fibre bundle strength or tenacity, g/tex; $F_{f}$ = breaking load of fibre, kg; x = load cell output, digital values and $W_{f}$ = weight of fibre sample, mg

### 3.3 Fibre fineness measurement unit

For developing the embedded unit to measure the fineness of the fibre, the air-flow method based on Kozney's theorem was employed [56,69]. According to Kozney's theorem, the specific surface or the total surface of fibre per unit volume is the measure of fibre fineness and can be expressed by equation (8) [56].

(8) $$S_{f}=\sqrt{\frac{\varepsilon \;.\Delta P}{Q_{a}}}$$

Where: $S_{f}$ = specific surface of fibre, mm^2^; ε = constant, ΔP = difference in pressure, dyn/mm^2^_; and_ $Q_{a}$ = rate of flow of air, mm^3^/s

Again, the fibre fineness (tex) may be expressed by equation (9).

(9) $$T\propto \frac{1}{{S_{f}}^{2}}\propto \frac{Q_{a}}{\Delta P}$$

So, if Δ*P* is kept constant, the flow rate ($Q_{a}$*)* will be proportional to the tex value (*T*) of the fibre.

**Fig. 4:**
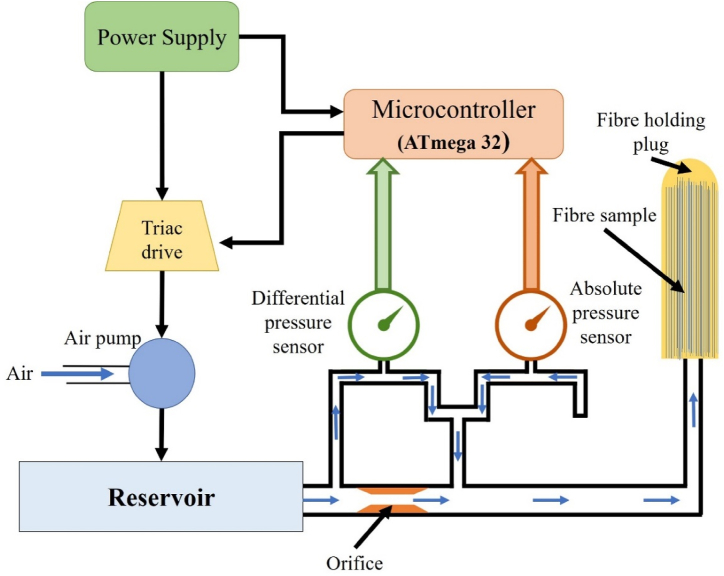
Block diagram of banana fibre fineness measurement unit.

The block diagram of the developed fibre fineness measurement unit is depicted in Fig. 4. In the developed unit, a pneumatic/air pump was used to pump the air to the plug-through reservoir. The air pump was operated by the traic circuit and relay switch to maintain the constant flow. The reservoir was used to store as well as maintain the constant flow of air without pressure drop in the circuit.

**Fig. 5:**
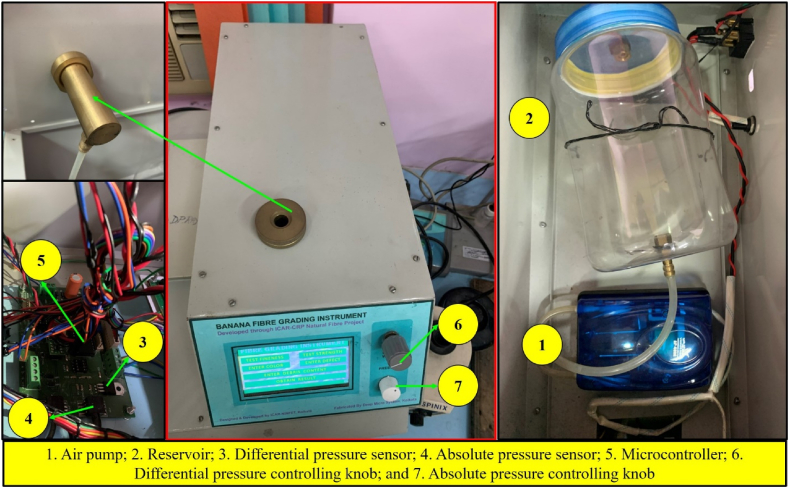
Hardware of the developed fibre fineness measurement unit.

To sense as well as maintain the pressure in the flow line, an absolute pressure sensor (MPXV7002) was used. An orifice was placed between the reservoir and fibre sample plug to create the variation in pressure, and this variation in pressure was sensed by the differential pressure sensor. This value of pressure was used to calculate the fineness of the fibre. Finaly, an algorithm was developed by using the C^++^ language to control of the operation of complete fineness measurement unit and convert the pressure difference reading into fibre fineness (tex). At last developed algorithm was uploaded on the microcontroller to execute the operation. Hardware of the developed fineness measurement unit is illustrated in Fig. 5.

### 3.4 Visual interface-based data acquisition unit

The developed data acquisition unit processed, computed, displayed and stored the quality assessment data. The unit included three sub-units, i.e., electrical circuit for receiving data, electrical circuit for storing data and HMI-assisted display. The electrical circuit of receiving data mainly consists of a master microcontroller (Arduino Mega ATmega 2560) which collects and processes the output signals of the fibre bundle strength and fineness measurement units and computes the overall quality of the banana fibre. After that the final results were simultaneously transferred to the electrical circuit of storing unit to store the data in TXT format and HMI-assisted display unit for visual presentation of output results in HMI-assisted display.

#### 3.4.1 Design and development of visual interface

**Fig. 6:**
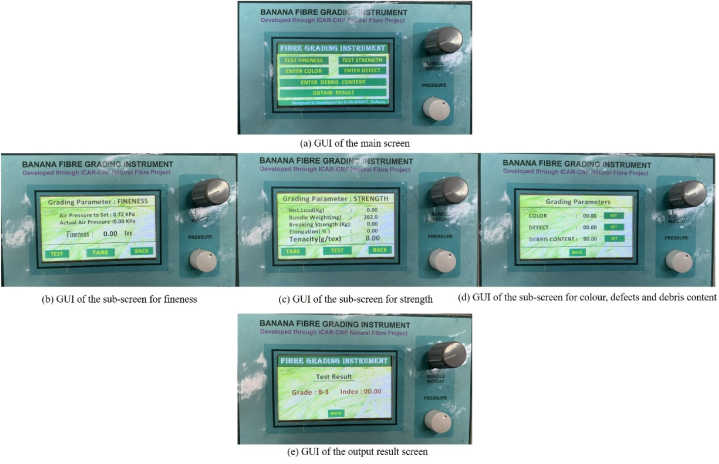
GUIs of the developed visual interface of banana fibre quality assessment instrument.

For designing the visual interface of the instrument, a Nextion enhanced (NX4827K043) 4.3-inch HMI Smart UART TFT touch display was used. The developed visual interface comprises multiple operator-friendly touch screens and each screen comprises a specially designed graphical user interface (GUI) for the visual presentation of the banana fibre quality assessing parameters. The designed visual interface comprises a ‘main screen’, three ‘sub-screens’ for the individual parameters and one ‘output screen’ as shown in Fig. 6 (a) to Fig. 6 (e) respectively. The GUI of each screen was designed and programmed by using the Nextion Editor software. The main screen of the developed visual interface consists of the digital switches for parameters i.e. fineness, strength, colour, defects, debris content and overall quality results. Each digital switch was programmed in such a way that as the operator gives the command to the Nextion display by touching the particular digital switch, then the developed algorithm processes the information and opens the particular parameter output screen.

The ‘strength’ sub-screen displays the value of fibre instant load (kg), bundle weight (mg), breaking strength (kg), elongation (%) and tenacity (g/tex). To initiate the bundle strength test, the measured value of the fibre bundle weight was entered. After that, ‘TEST’ switch was pressed to perform the test procedure. After the completion of test, value of bundle strength in terms of tenacity (g/tex) displayed on the screen. In addition to this, the screen also comprises the ‘BACK’ switch to go to the home screen and ‘TARE’ switch to zero the past readings and rerun the test procedure. The fineness sub-screen shows the reading of air pressure to set (kPa) and actual air pressure (kPa) and fineness (tex). Similar to the ‘strength’ sub-screen ‘fineness’ sub-screen also comprises the ‘BACK’ and ‘TARE’ digital switches. The ‘colour, defects and debris content’ sub-screen encompasses the provision to set colour (%), defects (% weight) and debris content (% weight) values by manual. Finally output screen of the developed instrument displays the overall quality of fibre on the basis of all five parameters. The screen also comprises the ‘BACK’ switch to go to the home screen for repeating the test.

#### 3.4.2 Development of data acquisition system

**Fig. 7:**
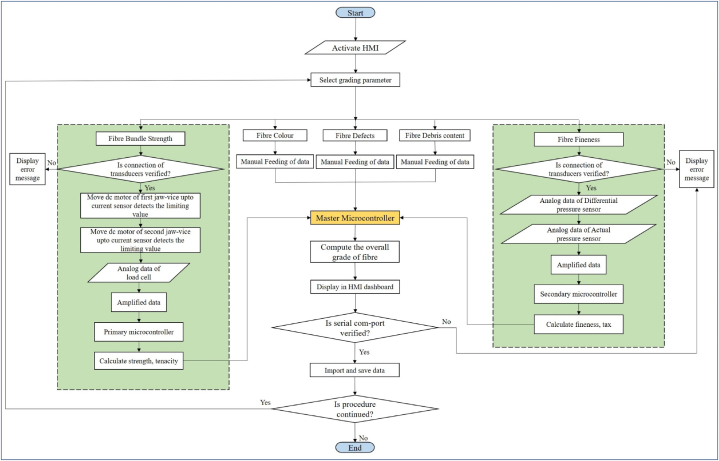
Flow diagram of the visual interface-cum-data acquisition system.

**Fig. 8:**
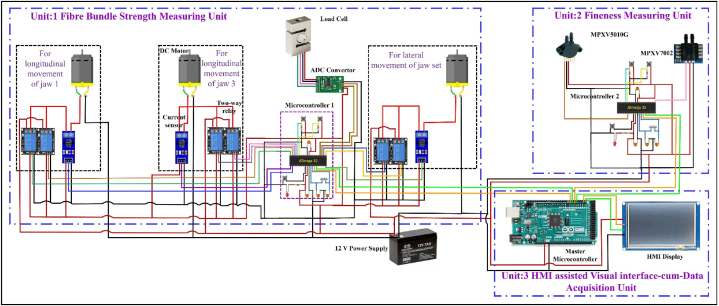
Overall circuit diagram of the developed banana fibre quality assessment instrument.

After developing the GUI of various screens, Nextion HMI display and SD card module were integrated to the master microcontroller (Arduino ATmega 2560) for displaying and storing the data respectively. The HMI display was connected to the microcontroller via TTL serial communication whereas SD card module was connected through SPI communication. The output signal of the embedded units developed for the ‘fibre bundle strength’ and ‘fineness’ measurement was connected to the microcontroller through the polyvinyl chloride cables. The algorithms developed for measuring the output value of fibre bundle strength and fineness were integrated into one algorithm and a final algorithm to measure the overall quality of fibre on the basis of five quality parameters was developed in Arduino IDE and uploaded to the master microcontroller. The developed algorithm executes the operation and measure the overall quality of fibre by following the procedure as described in Fig. 7. Finally, the hardware of the fibre bundle strength measurement unit, fineness measurement unit and visual interface as presented in Fig. 2, Fig. 4, Fig. 6 respectively was developed by following the overall circuit diagram as presented in Fig. 8.

### 3.5 Cost of the developed system

The approximate cost of the materials required for developing the instrument is given in Table 1. The toral cost required for developing the instrument is approximately $1800.00. The developed instrument is easy to build, affordable and low in cost and may be helpful for researchers, academia and stakeholders working in the field of processing of banana pseudo-stem fibre.

**Table 1:** Cost of the components required for developing the instrument.

S.N.	Component/item	Approximate price, $
1	**Fibre bundle strength measurement unit**	
i	Mechanical setup of bundle strength measurement unit	240
ii	DC motors and wiper motors	120
iii	Microcontroller	59.5
iv	Load cell	300
v	Accessories: ADC convertor, current sensors, two-way relay, resistor, capacitor, amplifier, limit switch, ramp generator, power supply, wires, etc.	250
	**Subtotal**	**969.5**
2	**Fibre fineness measurement unit**	
i	Mechanical setup of fineness measurement unit	200
ii	Air pump	35.5
iii	Microcontroller	59.5
iv	Pressure sensors	25.5
v	Accessories: resistor, capacitor, amplifier, voltage regulator, wires, etc.	150
	**Subtotal**	**470.5**
3	**Visual interface-based data acquisition unit**	
i	HMI Assisted display	180
ii	Microcontroller	90
iii	Accessories: PCB board, ICs, resistor, capacitor, amplifier, voltage regulator, wires, etc.	90
	**Subtotal**	**360**
	**Total**	**1800**

### 3.6 Collection of fibre samples

**Fig. 9:**
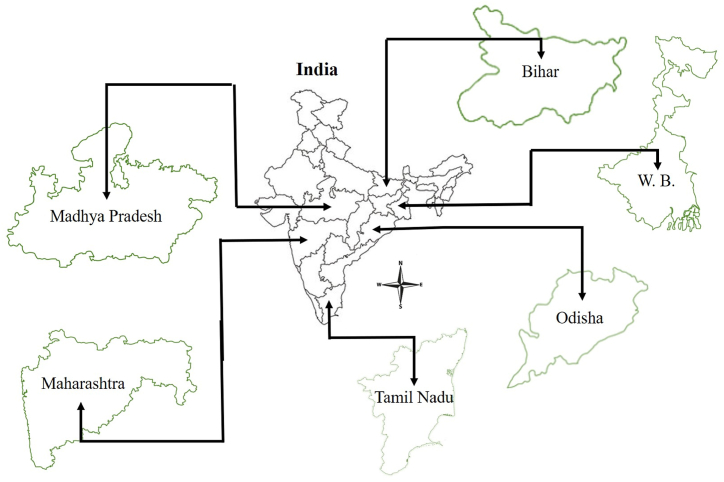
Collection of fibre samples from different states of the country.

A total of 150 kg (25 kg of each variety/cultivar) of fibre extracted from banana pseudo-stem of the following banana cultivars viz., Dwarf Cavendish, Basrai, Grand Naine, Rasthali, Robusta and Champa were collected from different states as mentioned in Fig. 9 and Table 2. The samples were collected during 2019-20 and 2022-23 years. The collected fibre was stored in a closed chamber at ambient temperature and relative humidity conditions. As per Table 2, a total of 180 samples from the combination of six varieties (cultivars), three sets of moisture content and fifteen replications were prepared and analyzed. The procedure for fibre quality assessment is described in subsequent section.

**Table 2:** Selection of different banana varieties and moisture content for experiment.

State	Variety (Cultivar)	Moisture Content (db), %
Bihar	V_1_: Dwarf Cavendish	M_1_: 6.00 to 6.99 M_2_: 7.00 to 7.99 M_3_: 8.00 to 8.99
Madhya Pradesh	V_2_: Basrai	
Maharashtra	V_3_: Grand Naine	
Odisha	V_4_: Rasthali	
Tamil Nadu	V_5_: Robusta	
West Bengal	V_6_: Champa	

### 3.7 Test procedure

To initiate the tests, the laboratory condition was maintained at 65 ± 2 % RH and temperature at 27 ± 2 °C. For minimizing the errors during tests, fibre samples were conditioned to a moisture equilibrium state in standard atmosphere conditions. After that, the strength and fineness of banana fibre were measured in the developed instrument by following the procedure inscribed in IS 7032-1: 1986 [61] with fifteen replications.

For measurement of fibre bundle strength, test sample of 125 mm of length and 150–250 mg of weight was separated from the middle portion of the fibre reed. These samples were prepared by randomly selecting the fibres from collected raw fibre bunch (∼25 kg). Subsequent test samples were placed in between the mechanical jaws of the fibre bundle strength measurement unit. To initiate the test, the measured value of the fibre bundle weight was entered through the HMI-assisted touch display-based visual interface. Thenceforth, ‘TEST’ switch was pressed to perform the test procedure. After the completion of test, value of breaking load in kg, elongation in percentage and bundle strength in tenacity (g/tex) was displayed on the HMI-assisted screen.

Similarly, for determination of fibre fineness, fifteen samples of 300–400 mg of fibre s were prepared form raw fibres. The samples were cleaned to make them free from specky and knotty spots, and hard gummy fibres. The fibre samples were placed into fibre plug of the fibre fineness measurement unit in such a way that fibres were uniformly accommodated inside the plug. The plug was then placed into the fineness port of the instrument. After that a value of pressure of 0.72 kPa was set through the pressure knob located near the visual interface [56]. Hereafter, test was run by pressing the digital switch ‘TEST’ provided in the ‘Fineness’ sub-screen of the visual interface. After the termination of the test, the visual interface displayed the fineness of the fibre in ‘tex’.

For the overall quality of the fibre, colour, defects and debris were measured by ‘hand and eye method’ and instrumentation method as mentioned in IS: 271 2020 [60] and IS 7032: 1986 [61]. These measured values were inserted into the ‘Colour, defects and debris content’ sub-screen of the visual interface. Subsequently, ‘Obtain results’ digital switch was executed to get the overall quality of the fibre. Once the ‘Obtain results’ digital switch was pressed it opens the ‘Test results’ sub-screen and displayed the overall quality of the fibre. Finally, the developed algorithm automatically logged the values of each parameter and overall quality of the fibre in the microSD card. At last, test was completed with pressing of ‘BACK’ digital switch.

## 4 Results and discussion

### 4.1 Fibre bundle strength

The developed instrument was rigorously evaluated in the laboratory to examine the accuracy of fibre bundle strength measurement unit. The laboratory evaluation informed that the developed bundle strength measurement unit can measure the bundle strength between 0 and 250 g/tex. Statistically analyzed results as presented in Table 3, show that the mean value of bundle strength varied from 20.92 g/tex to 28.31 g/tex whereas, the coefficient of non-uniformity (CNU) varied from 0.14 % to 3.10 % in different treatments. The bundle strength of fibre measured by the developed instrument was also compared with the values measured by the conventional (manual) method and it was observed that the mean absolute percentage error (MAPE) was found in the range of 0.62 %–4.96 %. The standard mean error (SME) in the measured values of bundle strength by the conventional method and developed instrument was found as 0.34 and 0.35 respectively. The small range of CNU and MAPE indicates that the developed instrument exhibits a high degree of accuracy and precision in the measurement of fiber bundle strength. The findings of the tests confirm that the developed system exhibits a high degree of accuracy and precision in measuring bundle strength, displaying minimal deviation from the actual values. A similar kind of acceptance of CNU and MAPE of values measured by the embedded system was also reported by Shrivastava et al. (2023) [70], Shrivastava et al. (2023) [71], Shafaei et al. (2019) [72] and Singh et al. (2021) [73].

**Table 3:** Statistical analysis of obtained data of fibre bundle strength and fineness measured through developed system.

Treatment	Bundle Strength					Fineness				
	Mean, g/tex	SD	CV	CNU, %	MAPE, %	Mean, tex	SD	CV	CNU, %	MAPE, %
**V**_**1**_**M**_**1**_	25.09	0.44	1.76	1.35	0.62	6.09	0.14	2.34	1.78	9.76
**V**_**1**_**M**_**2**_	23.49	0.08	0.32	0.25	2.44	6.05	0.06	0.95	0.73	2.33
**V**_**1**_**M**_**3**_	20.92	0.22	1.06	0.79	1.88	5.99	0.04	0.59	0.45	0.98
**V**_**2**_**M**_**1**_	26.03	0.28	0.01	0.79	2.25	6.26	0.04	0.70	0.54	1.97
**V**_**2**_**M**_**2**_	25.55	0.07	0.27	0.20	1.15	6.35	0.10	1.61	1.15	1.44
**V**_**2**_**M**_**3**_	24.96	0.47	1.87	1.28	2.93	6.05	0.18	3.03	2.29	3.98
**V**_**3**_**M**_**1**_	24.64	0.49	1.99	1.53	1.67	6.20	0.07	1.20	0.90	0.71
**V**_**3**_**M**_**2**_	24.25	0.04	0.18	0.14	0.73	6.33	0.15	2.37	1.70	6.64
**V**_**3**_**M**_**3**_	22.95	0.31	1.34	0.97	1.06	5.77	0.75	12.93	9.95	9.49
**V**_**4**_**M**_**1**_	24.52	0.52	0.02	1.61	1.81	6.41	0.09	1.34	0.92	8.77
**V**_**4**_**M**_**2**_	24.35	0.80	3.30	2.53	3.57	6.34	0.16	2.50	6.12	5.71
**V**_**4**_**M**_**3**_	22.93	0.61	2.65	2.04	0.02	6.32	0.07	1.09	0.81	6.02
**V**_**5**_**M**_**1**_	24.40	0.35	1.45	1.10	1.62	6.21	0.03	0.48	0.36	3.14
**V**_**5**_**M**_**2**_	23.08	0.93	4.05	3.10	4.96	6.34	0.07	1.05	0.74	3.43
**V**_**5**_**M**_**3**_	22.78	0.77	3.39	2.48	2.61	6.30	0.03	0.43	0.33	0.68
**V**_**6**_**M**_**1**_	28.31	0.68	2.41	1.62	3.34	5.70	0.50	8.70	6.66	13.62
**V**_**6**_**M**_**2**_	27.02	0.08	0.31	0.24	1.17	5.82	0.49	8.44	6.08	10.82
**V**_**6**_**M**_**3**_	27.29	0.14	0.52	0.40	1.63	5.63	0.56	9.98	7.58	10.31

**Fig. 10:**
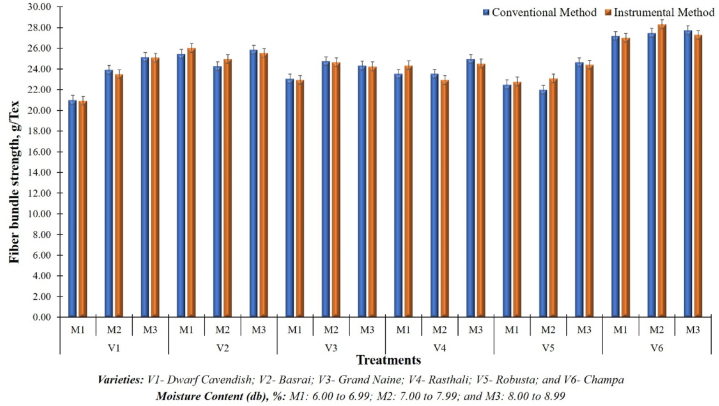
Comparison between bundle strength measured by developed instrument with conventional method.

The obtained results were further statistically analyzed using the one wayANOVA with DMRT post hoc test. The results presented in Table 4, established a non-significance difference between the bundle strength values measured by the developed instrument and conventional method at a 5 % level of significance(*One-Way ANOVA, F*_*28,2*_ = *3.914, P* = *0.224*). It shows that the measured values are at par with the actual values of bundle strength. A comparative graph of fibre bundle strength measured by the developed instrument and conventional method is depicted in Fig. 10. The highest value of bundle strength of 28.31 g/tex was observed at V_6_M_1_ (V: Champa and MC: 6.00–6.99 %) treatment while the lowest value of 20.92 g/tex was observed at V_1_M_3_ treatment (V: Dwarf Cavendish and MC: 8.00–8.99 %). Based on the results it can be concluded that the ‘Champa’ variety of banana produces a superior quality of fibre compared to other selected varieties in terms of fibre bundle strength.

**Table 4:** Comparison between bundle strength measured by developed instrument with conventional method.

	Sum of Squares	df	Mean Square	F	Sig.
Between Groups	106.752	28	3.813	3.914	0.224
Within Groups	1.948	2	0.974		
Total	108.701	30

The obtained results were further analyzed to examine the effect of variety and moisture content on bundle strength of fibre. The perusal of data as presented in Table 5 revealed that both variety and moisture content have individual as well as combined effects on fibre strength at a 5 % level of significance (P < 0.001). It was observed that the strength of the bundle is inversely proportional to the moisture content present in the fiber. Changes in the value of bundle strength with different varieties of banana were observed, possibly due to differences in soil and agro-climatic variations in different regions [74,75]. Shivashankar et al., 2006 [76] also reported a significant difference in the tenacity (bundle strength) of fibre with different varieties. In this context, Mukherjee and Satyanarayana (1986) [77] analyzed that the quality matrix of fibre composed mainly of lignin and hemicellulose, varies in different cultivars (variety), which strongly influences the bundle strength of the fibre. Similarly, Taj et al. (2007) [78] strongly emphasized that lignocellulosic constituents contribute to the overall property of plant fibres.

**Table 5:** Effect of banana plant variety and moisture content on bundle strength of fibre.

Source	Type III Sum of Square	Df	Mean Square	F	Sig.
Corrected model	168.90	17	9.93	41.93	<0.001
Intercept	32646.66	1	32646.66	137461.80	<0.001
Variety	124.13	5	24.82	104.53	<0.001
Moisture Content	12.46	2	6.23	26.24	<0.001
Variety × Moisture Content	32.30	10	3.23	13.59	<0.001
Error	8.55	36	0.237		
Total	32824.12	54			

### 4.2 Fibre *fineness*

**Fig. 11:**
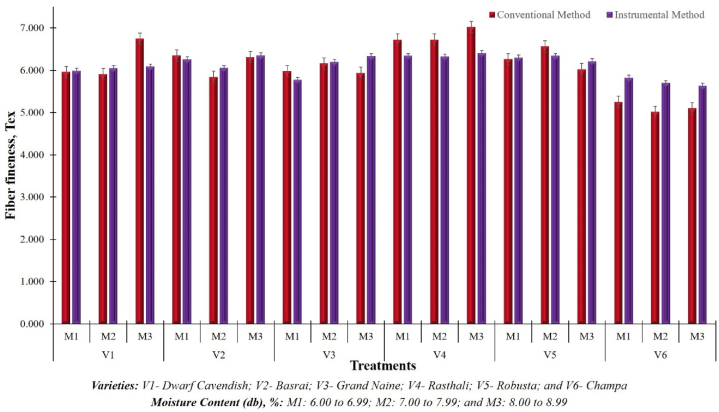
Comparison between fineness measured by developed instrument with conventional method.

In the same manner as fibre bundle strength was tested, the fineness of the fibre was also measured by the developed instrument and compared with the fineness measured by the conventional (manual) method. The laboratory evaluation indicated that the developed unit is able to measure the fineness of fibre ranging from 0 to 25 tex. The statistical comparison of the data as summarized in Table 2, showed that, fineness of the fibre varied from 5.63 tex to 6.41 tex in different treatments whereas coefficient of non-uniformity (CNU) varied from 0.33 % to 9.95 %. The mean absolute percentage error (MAPE) of banana fibre fineness measured by the developed instrument over the values measured by the conventional (manual) method was found in the range of 0.68 %–13.62 %. Both MAPE and CNU data sets in case of fibre fineness were varied because of the variation in the plant varieties and the prevailing temperature condition. A comparative graph of fineness measured by the developed instrument and conventional method is depicted in Fig. 11. The study results showed that the lowest value of fineness of 5.63 tex was observed in case of V_6_M_3_ (V: Champa and MC: 8.00–8.99 %) treatment while the highest value of 6.41 tex was observed in case of V_4_M_1_ (V: Rasthali and MC: 6.00–6.99 %) treatment. Based on the results it can be concluded that the ‘Champa’ variety of bananas produces the finest quality of fiber among the selected varieties. From a textile application perspective, fibre having lower fineness values are preferable as this quality of fibre can be utilized either solely [79] or in blend with other natural fibers [[80], [81], [82]] for yarn and fabric production. On the other hand, coarse fibre (lower value of fineness) can be utilized for the production of nonwoven fabrics [[83], [84], [85], [86]].

The analysis of variance (ANOVA) with DMRT post hoc test results as presented in Table 6 indicates that there is no significant difference between the fineness values obtained from the developed instrument and those obtained using the conventional method at a 5 % level of significance (*One-Way ANOVA, F*_*51,2*_ = *4.730, P* = *0.190*). e The results signified that the measured values are in close range to the actual values of fineness.

**Table 6:** Comparison between fibre fineness measured by developed instrument with conventional method.

	Sum of Squares	df	Mean Square	F	Sig.
Between Groups	6.062	51	0.119	4.730	0.190
Within Groups	0.050	2	0.025		
Total	6.112	53			

The effect of variety and moisture content on fibre fineness were also analyzed by using the DMRT post hoc test in SPSS (Student version) software and presented in Table 7. The ANOVA results revealed that only variety has a significant effect on fibre fineness at a 5 % level of significance (P < 0.001), whereas moisture content and the combination of both variety and MC has a non-significant effect on fibre fineness. Preethi and Murthy (2013) [19] and Shivashankar et al. (2006) [76] also experienced a similar trend of variation in fineness with cultivars in their findings. Balakrishnan et al. (2019) [40] and Balakrishnan et al. (2020) [87] also conducted a similar study to examine the variation in fibre fineness with Sri Lankan banana cultivars, their results revealed that cultivars have a most significant influence on fibre fineness. Similar to the bundle strength, variation in fineness with different varieties was observed due to the difference in soil and agro-climatic variation in different regions. Since the fineness is an inherent property of the fibre, therefore, external factors i.e. MC and temperature have a minimal or no influence on fibre fineness. Therefore, a non-significant relation between the MC and fibre fineness was established through this study. Balakrishnan et al. [40], and Venkateshwaran et al. (2011) [88], also studied the effect of moisture content on mechanical properties of banana fibre/epoxy composite. They reported that moisture content affected the fineness but not significantly.

**Table 7:** Effect of banana plant variety and moisture content on fineness of fibre.

Source	Type III Sum of Square	Df	Mean Square	F	Sig.
Corrected model	3.109	17	0.183	2.192	0.024
Intercept	2022.479	1	2022.479	24245.389	<0.001
Variety	2.350	5	0.470	5.635	<0.001
Moisture Content	0.078	2	0.039	0.465	0.632
Variety × Moisture Content	0.681	10	0.068	0.817	0.615
Error	3.003	36	0.083		
Total	2028.591	54			

### 4.3 Overall quality of fibre

As there is no standard method for quality assessment of banana fibre is available in the Indian textile sector, therefore, based on well-established grading system of jute fibre recognized by Indian Standard [60], a categorization system was introduced in this research article to categorize the collected samples and presented in Table 8. The collected samples were categorized into four major categories in addition to the reference category in the same manner as mentioned in IS: 271 2020 [60] for jute fibre.

**Table 8:** Categorization of banana fibre for overall assessment of quality.

Category	Strength (g/tex)	Fineness (tex)	Debris content (wt%)	Colour, (%)	Defect, (Wt%)	Overall Score
**Reference**	Score: 40 (High) Range: ≥25.0	Score: 24 (Very fine) Range: ≤4.0	Score: 19 (Very low) Range: ≤5.0	Score: 11 (Excellent) Range: ≥65.0	Score: 6 (Very low) Range: ≤0.25	**100**
**BN-1**	Score: 32 (Medium) Range: 24.9–20.0	Score: 19 (Fine) Range: 4.1–4.9	Score: 15 (Low) Range: 5.1–9.0	Score: 9 (Good) Range: 64.9–55.0	Score: 5 (Low) Range: 0.26–0.50	80
**BN-2**	Score: 24 (Medium) Range: 24.9–20.0	Score: 14 (Fine) Range: 4.1–4.9	Score: 11 (Low) Range: 9.1–13.0	Score: 7 (Good) Range: 54.9–45.0	Score: 4 (Low) Range: 0.51-0.75	60
**BN-3**	Score: 16 (Average) Range: 19.9-15.0	Score: 9 (Course) Range: 5.0–5.9	Score: 7 (Medium) Range: 13.1–17.0	Score: 5 (Average) Range: 44.9–35.0	Score: 3 (Medium) Range: 0.76–1.00	40
**BN-4**	Score: 8 (Poor) Range: ≤14.9	Score: 4 (Very course) Range: ≥6.0	Score: 3 (High) Range: ≥17.1	Score: 3 (Poor) Range: ≤34.9	Score:2 (High) Range: ≥1.01	20

The reference category of fibre represents the ideal or best quality of the fibre in terms of all parameters i.e. strength, fineness, debris content, colour and defects. Hence, the highest score for all five quality parameters i.e. strength is equal to 40 (≥25.0 g/tex), fineness is equal to 24 (≤4.0 tex), debris content is equal to 19 (≤5.0 %), colour is equal to 11 (≥65.0 %) and defects is equal to 6 (≤0.25 %) were assigned in this category to make the cumulative score of 100. In the same manner, four different categories namely BN-1, BN-2, BN-3 and BN-4 were formulated and proportional score were assigned as mentioned in Table 8. According to the mentioned classification of fibre, ‘reference’ represents the ‘excellent’, with a cumulative score of 100 ‘BN-1’ represents the ‘very good’, with a cumulative score of 80 ‘BN-2’ represents the ‘good’, with a cumulative score of 60 ‘BN-3’ represents the ‘average’ with a cumulative score of 40 and ‘BN-4’ represents the ‘poor’ quality of fibre with a cumulative score of 20. The score and range of each parameter were selected based on stakeholders' feedback, market survey, and scientific reports on jute fibre [60,68]and comprehensive testing of fibre qualities through this study.

On the basis of, proposed score and range of each parameter as presented in Table 8, the overall grade of the banana fibre was measured by the developed system and compared with the quality of the fibre measured by the conventional method. The overall quality of the fibre measured in terms of score was analyzed statistically using the independent sample T-test in SPSS software and presented in Table 9. The presented data, enunciated that there is no significant difference was established between the fibre quality measured by developed instrument with conventional method at 5 % level of significance. It signifies that the developed instrument was quite precise as well as accurate in measuring the overall quality of fibre with little deviation from the true results (conventional method). A comparison between the overall quality of fibre measured by the developed instrument and the conventional method as prevailing in jute grading system [58,60]is presented in Table 10. The tabulated results indicated that, during validation of the developed system, only three treatments (**V**_**3**_**M**_**2,**_ **V**_**5**_**M**_**1,**_ **and V**_**5**_**M**_**3**_) showed a significant variation in the overall grade of fibre whereas, in rest of treatments, almost similar grade of fibre was observed. The processing time of the developed system for measuring the fibre bundle strength, fineness and overall grade of one sample of banana fibre was observed as 2.5 s whereas the total processing time (sample preparation time + processing time of the developed system) of one sample was observed as 4.8 min. Hence, the total sampling rate of the developed system was observed as approximately 12 samples/h. Finally, it can be concluded that the developed system is an accurate, feasible and time-ordered technique for the assessment of the overall quality of the banana fibre and it is well suited for the actual conditions. The application of the developed system in textile industries may increase the adaptation of banana fibre in the future and also reduce the dependency in other natural fibres.

**Table: 9:** Categorization of banana fibre for overall assessment of quality.

		F	Sig.	t	df	Sig. (2-tailed)	Mean Difference	Std. Error Difference	95 % Confidence Interval of the Difference	
									Lower	Upper
Overall quality of fibre	Equal variances assumed	0.95	0.33	3.61	34	0.001	6.87	1.89	3.01	10.73
	Equal variances not assumed	–	–	3.61	32.49	0.001	6.87	1.89	3.00	10.73

**Table 10:** Comparison of the quality of the banana fibre measured by developed instrument and conventional method.

Treatment	Conventional Method	Instrumental Method
**V**_**1**_**M**_**1**_	BN-2 + 20 % higher	BN-2 + 33.5 % higher
**V**_**1**_**M**_**2**_	BN-2	BN-2
**V**_**1**_**M**_**3**_	BN-2 + 50 % higher	BN-2 + 40 % higher
**V**_**2**_**M**_**1**_	BN-2 + 70 % higher	BN-2 + 65 % higher
**V**_**2**_**M**_**2**_	BN-2	BN-2
**V**_**2**_**M**_**3**_	BN-2 + 50 % higher	BN-2 + 40 % higher
**V**_**3**_**M**_**1**_	BN-2 + 35 % higher	BN-2 + 46.5 % higher
**V**_**3**_**M**_**2**_	BN-2 + 10 % higher	BN-3 + 86.5 % higher
**V**_**3**_**M**_**3**_	BN-2 + 25 % higher	BN-2
**V**_**4**_**M**_**1**_	BN-3 + 85 % higher	BN-3 + 86.5 % higher
**V**_**4**_**M**_**2**_	BN-2	BN-2
**V**_**4**_**M**_**3**_	BN-2 + 20 % higher	BN-2
**V**_**5**_**M**_**1**_	BN-2 + 10 % higher	BN-3 + 86.5 % higher
**V**_**5**_**M**_**2**_	BN-2 + 30 % higher	BN-2 + 13.5 % higher
**V**_**5**_**M**_**3**_	BN-2	BN-3
**V**_**6**_**M**_**1**_	BN-2 + 90 % higher	BN-2 + 86.5 % higher
**V**_**6**_**M**_**2**_	BN-2 + 80 % higher	BN-2 + 73.5 % higher
**V**_**6**_**M**_**3**_	BN-2 + 70 % higher	BN-2 + 86.5 % higher

## 5 Conclusion

Assessment of fibre quality of natural fibre remained ever challenging for depicting its true-value in real market. Banana fibre, being minor in the category of natural fibres has faced the similar problems to establish its ingenuity for its value-chain establishment. The low yield from huge biomass, the coarseness of fibre and non-uniform availability made the task much difficult as there has been no standard instrument available to measure its basic quality parameters i.e. strength and fineness for textile application. To address the issue an attempt has been made to develop a novel sensor-based digital instrument for assessing the aforesaid quality parameters and overall grade of fibre extracted from banana pseudo-stem. Developed instrument mainly consists of a fibre bundle strength measurement unit, fineness measuring unit and visual interface cum data acquisition unit. Laboratory evaluation of developed instrument revealed that bundle strength and fineness measured by developed instrument varied from 20.92 g/tex to 28.31 g/tex and 5.63 tex to 6.41 tex respectively in different treatments. Furthermore, test results of the developed system showed a good correlation between the measured and actual outputs with a non-significance difference between the values of bundle strength(*One-Way ANOVA, F*_*28,2*_ = *3.914, P* = *0.224*), fineness (*One-Way ANOVA, F*_*51,2*_ = *4.730, P* = *0.190*). and overall quality of fibre (*Independent sample T-Test, F*_*34,1*_ = *0.95, P* = *0.190*). at 5 % level of significance. The developed instrument is easy to build as well as easy to use and have an approximate cost of $1800.00. The present study also introduced a grading system for quality assessment of banana fibre based on the well-established and well-recognized grading system of jute fibre developed by Indian Standard (IS: 271 2020). The findings of the present study, concluded that the combination of developed instrument and grading system is an accurate, feasible and time-ordered technique for the assessment of the overall quality of the banana fibre and well suited for the actual conditions. The implementation of present study in Indian textile industries may increase the adaptation of banana fibre for value-added product development and promote the concept of circular economy amongst the Indian banana-growing farmers in the future.

## Source of funding

No funding was received for the publication of this manuscript.

## Data availability statement

Data will be made available on request.

## CRediT authorship contribution statement

**Deb Prasad Ray:** Writing – review & editing, Writing – original draft, Investigation, Conceptualization. **Prateek Shrivastava:** Writing – review & editing, Writing – original draft, Methodology, Data curation, Conceptualization. **Rakesh Kumar Ghosh:** Writing – review & editing, Validation, Data curation. **Manik Bhowmick:** Validation, Methodology, Investigation. **D.B. Shakyawar:** Writing – review & editing, Writing – original draft, Conceptualization. **Ipsita Das:** Investigation, Formal analysis, Data curation. **Gunasindhu Sardar:** Methodology, Investigation, Formal analysis. **Jayanta Mondal:** Formal analysis, Data curation. **S.C. Saha:** Validation, Investigation, Formal analysis. **Gautam Roy:** Visualization, Validation, Methodology, Data curation.

## Declaration of competing interest

The authors declare that they have no known competing financial interests or personal relationships that could have appeared to influence the work reported in this paper.
